# The Temporal Experience of Pleasure Scale (TEPS): Measurement Invariance Across Gender in Chinese University Students

**DOI:** 10.3389/fpsyg.2019.02130

**Published:** 2019-09-19

**Authors:** Huan Zhou, Wanting Liu, Jie Fan, Jie Xia, Jiang Zhu, Xiongzhao Zhu

**Affiliations:** ^1^Medical Psychological Center, The Second Xiangya Hospital, Central South University, Changsha, China; ^2^Medical Psychological Institute of Central South University, Changsha, China

**Keywords:** measurement invariance, anhedonia, TEPS, gender, confirmatory factor analysis

## Abstract

The Temporal Experience of Pleasure Scale (TEPS) is a self-report instrument assessing pleasure experience. The present study aimed to confirm the factor model of the Chinese version of TEPS and test measurement invariance of the scale across gender in Chinese university students. Participants were 2977 (51% female) undergraduates aged from 16 to 27 years (Mean age = 18.9 years). Results indicated that the revised four-factor structure of the TEPS had acceptable fit in the total sample and in gender groups. Furthermore, configural, metric and partial scalar invariance across gender were established. Two of the items (item 4 and 8) demonstrated different intercepts and women scored higher than men on both items. With partial scalar invariance demonstrated, test of differences in latent means indicated that men had lower levels of pleasure when compared with women. To our knowledge, this study is the first attempt to test the measurement invariance of the TEPS across gender, which provides support for future research that involves examining hedonic capacity in Chinese men and women.

## Introduction

Anhedonia, a reduced ability to experience pleasure in normally pleasurable situations (Harvey et al., 2007), is one of the negative symptoms of schizophrenia and is also a critical feature of depression related to social functioning in these patients (Kelley et al., 1999; Keshavan et al., 2011). Some studies have recently suggested that trait anhedonia can be identified in a non-clinical sample of young adults (Chan et al., 2012b; Li Z. et al., 2015; Wu et al., 2017). With the deepening research of anhedonia, studies both in animal and in humans (Berridge and Robinson, 2003; Kringelbach and Berridge, 2009) have indicated that pleasure experience is not a unitary construct, but can be parsed into two distinct subcomponents, namely anticipatory (or wanting) and consummatory (or liking) pleasure (Hollenbeck and Klein, 1987; Berridge and Robinson, 1998; Berridge and Kringelbach, 2008). The anticipatory pleasure factor captures the future-oriented pleasure, while the consummatory pleasure factor captures in-the-moment pleasure. To specifically capture these two distinguishing components, Gard et al. (2006) developed the Temporal Experience of Pleasure Scale (TEPS) based on theoretical models of anticipatory and consummatory pleasure. As they hypothesized, results in several large college-age samples supported the two-factor construct of trait disposition in experiences of pleasure. Subsequently, the TEPS has been widely used to measure hedonic capacity not only in people with psychosis-spectrum disorders (Mote et al., 2014; Yang et al., 2018) but also in healthy individuals (Da Silva et al., 2017; Li et al., 2019). It was also used in different countries such as America (Novacek et al., 2016), China (Li Y. H. et al., 2015), German (Simon et al., 2018), the United Kingdom (Ho et al., 2015), Spain (Gooding et al., 2016), and Australia (Geaney et al., 2015). All results showed adequate reliability and validity of the TEPS to assess the construct of pleasure. In addition, the measurement invariance of the TEPS across culture and time were examined (Li et al., 2018), supporting for its application. In 2012, Chan and colleagues examined the structure of TEPS in the Chinese context, with results finding that a four-factor model fit best in Chinese population. In this four-factor structure, the original two constructs of anticipatory and consummatory pleasure experience are further divided into abstract and contextual components. The abstract component refers to belief-oriented statements, whereas the contextual component refers to event-oriented statements. In other words, the Chinese version of TEPS consists of four distinct components, that is abstract anticipatory pleasure, contextual anticipatory pleasure, abstract consummatory pleasure, and contextual consummatory pleasure (Chan et al., 2012a; Li Z. et al., 2015).

Previous studies have suggested significant gender effects on pleasure or anhedonia. Several studies found that women seem to have higher level of pleasure experience than men. For example, Seidlitz and Diener (1998) revealed that women recalled positive events and memories more vividly than men. Gross and John (1995) found that women reported more positive emotions than men. Carver and White (1994) reported that women had higher trait reward responsiveness than men. While some research found that males demonstrated higher level of anhedonia than females, such as physical, anticipatory and consummatory anhedonia than females (Miller and Burns, 1995; Oishi et al., 2001; Gard et al., 2006). It is obvious that those gender differences in anhedonia have typically been reported based on observed scores using a mean difference method. However, there is ambiguity regarding whether differences in self-rated scale score means can be attributed to authentic trait differences between gender. Stated differently, without evidence to support measurement invariance, any inferences about gender differences are necessarily weak (Vandenberg and Lance, 2000).

To our best knowledge, validation of the measurement invariance of the Chinese version of TEPS across gender has not been carried out in any study. Demonstrating measurement invariance of the Chinese version of TEPS between male and female, will allow future researchers to use this scale confidently irrespective of the gender of adults under examination. Hence, the goals of this study are to examine the factor model of the TEPS and to test measurement invariance for the better-fitting factor model across gender in Chinese university students. If the scale is found to be invariant across gender, latent means will be tested to examine potential differences for men and women.

## Materials and Methods

### Procedures

Participants came from three universities of Hunan province in China between June and December, 2018. Convenient sampling techniques was used to recruit participants. Students were informed of the purpose and procedure of participating in this study through digital advertisement delivered by their counselor, who also helped to arrange the specific investigation time. Data were collected on paper during regular class break with two trained psychology student nearby. After signing the written informed consent, participants completed and returned the anonymous self-report questionnaires immediately. Ethics approval for this study was obtained from the Ethics Committee of the Second Xiangya Hospital of Central South University.

### Participants

Of 3066 undergraduates who agreed to take part in the study, 17 (0.6%) participants answered less than 90% of all items of the TEPS, 62 (2.0%) participants provided a same answer to every item and 10 (0.3%) participants did not report gender, and were thus eliminated. The final analytic sample consisted of 2977 undergraduates, and was a nearly even mix of men (1456) and women (1521). Participants aged between 16 and 27 years old. The mean (standard deviation, SD) ages of the men and women were 18.87 (0.93) years and 18.94 (1.224) years, respectively.

### Instruments

#### The Chinese Version of the Temporal Experience of Pleasure Scale

The TEPS (Gard et al., 2006) is a self-report instrument that assesses pleasure experience. The Chinese version of TEPS (Chan et al., 2012a) was used in this study. It is a revised version of the original 18-item scale, containing two additional items (“I love it when a baby snuggles into my arms” and “On the way to my first date with my beloved, I can hardly wait to see him/her”). According to Chan et al.’s (2012a) study, the Chinese version of TEPS consisted of four factors. Anticipatory abstract pleasure reflected the pleasure experienced in anticipation of something more abstract or broad in nature. Anticipatory contextual pleasure referred to pleasure experienced in anticipation of something that is more concrete in nature. Consummatory abstract pleasure was related to consummation of emotional experience of something that is more abstract in nature. And consummatory contextual pleasure was about consummation of emotional experience of something that is more concrete in nature. Participants response on a 6-point Likert scale rated from 1 (very false for me) to 6 (very true for me). A lower score indicates a higher anhedonia propensity. In this study, Cronbach’s alpha of the whole scale was 0.84, showing high internal consistency.

### Data Analysis

All analyses were conducted using Mplus Version 7.3 (Muthén and Muthén, 1998–2012). Firstly, a series of confirmatory factor analysis (CFA) were performed to examine the two- and four-factor models in the total sample. By comparing the goodness-of-fit for these models the better dimensional structure of the TEPS in Chinese university students was determined. And then, CFA of the better model was performed in male and female samples simultaneously. As the assumption of normality was not supported based on the Shapiro-Wilk test (*p* < 0.001), all the measurement models were estimated using the maximum likelihood estimation with standard errors and mean-adjusted Chi-square test statistics (Brown, 2006; Dietrich et al., 2013; Petrowski et al., 2018). Since the χ^2^ statistic is sensitive to sample size, it was not used as the crucial index. This study used several other goodness-of-fit indices to evaluate the fit of the models to the data, including chi-square/degree of freedom (χ^2^/df), comparative fit index (CFI), Tucker–Lewis index (TLI), the Bayesian information criterion (BIC), and root mean square error of approximation (RMSEA). For χ^2^/df, values around 5 and lower indicate an acceptable or good fit of the model. The CFI and TLI values higher than 0.90 and 0.95 are suggesting acceptable and excellent fits to the data, respectively. In relation to RMSEA indices, values lower than 0.08 demonstrate overall acceptable fit, and values lower than 0.05 demonstrate excellent fit (Hu and Bentler, 1995; Kline, 2010). In addition, the 90% confidence interval of the RMSEA was examined: the upper bound of this confidence interval less than 0.10 are taken to indicate acceptable fit (Rossi et al., 2010).

After identification of the optimal model of the TEPS, several multiple-group confirmatory factor analyses were carried out to test the measurement invariance of this scale across gender. Because full configural invariance, partial metric or scalar invariance are necessary to compare latent means, this study examined configural invariance, metric invariance, and scalar invariance. The three nested steps (Chen, 2007; Little et al., 2007; van de Schoot et al., 2012) were conducted progressively: configural invariance to assess whether the factor structure is same across independent samples; metric invariance to test the model fit when the factor loading of variables are constrained to be equal in men and women; scalar invariance to examine the equivalence of items’ intercepts across gender. Statistically, the more restrictive model will be tested only after the invariance of the less restrictive model is confirmed.

When comparing relative fit of nested models, the evidence of invariance is ΔCFI ≤ 0.01 and a smaller BIC value (Cheung and Rensvold, 2002). A small difference between two nested models may lead to a significant change of χ^2^ when the sample size is large, so this study didn’t rely on the chi-square difference test. If partial scalar invariance is supported, the test of latent mean differences between male and female groups will be performed (Baumgartner and Steenkamp, 1998; Vandenberg and Lance, 2000; Cheung and Rensvold, 2002).

## Results

### Descriptive Statistics

Descriptive data and gender differences on scale scores are presented in Tables 1, 2. The TEPS total scores ranged from 25 to 120 (25–120 among males and 29–120 among females), with a mean (SD) of 77.75 (14.05) for males and 84.23 (14.63) for females. Sex difference on the total scale scores was statistically significant (μ = −12.255, *p* < 0.001). The present study observed that in the four-factor model, the female participants reported significantly higher scores than the male participants on contextual anticipatory pleasure, abstract consummatory pleasure, and contextual consummatory pleasure, while there was no significant difference on abstract anticipatory pleasure. In the two-factor model, men reported higher level of anhedonia than women, both on anticipatory and consummatory pleasure. Table 3 shows correlation of the 20 items of the Chinese version of TEPS. Table 4 shows the correlations among the factors in the total sample and separately for both gender groups. All correlations were statistically significant.

**TABLE 1:** Descriptive data and gender differences on factors of TEPS.

		***N***	**Mean**	**SD**	**μ**	***p***
**Four factors**						
AA	Men	1456	19.27	3.66	–1.866	0.062
	Women	1521	19.58	3.43		
CA	Men	1456	13.95	4.61	–16.924	<0.001
	Women	1521	16.91	4.64		
AC	Men	1456	21.82	4.92	–8.331	<0.001
	Women	1521	23.33	4.31		
CC	Men	1456	18.96	5.20	–6.382	<0.001
	Women	1521	20.18	4.93		
**Two factors**						
Anticipatory	Men	1456	38.50	7.73	–11.469	<0.001
	Women	1521	41.82	7.71		
Consummatory	Men	1456	35.50	7.64	–9.383	<0.001
	Women	1521	38.18	7.23		
Total	Men	1456	77.75	14.05	–12.255	<0.001
	Women	1521	84.23	13.63		

*AA, abstract anticipatory pleasure; CA, contextual anticipatory pleasure; AC, abstract consummatory pleasure; CC, contextual consummatory pleasure; ANT, anticipatory pleasure; CON, consummatory pleasure.*

**TABLE 2:** Descriptive data and gender differences on items scores.

**Item**	**Sex**	***N***	**Mean**	**SD**	**μ**	***p***
1	Men	1456	2.98	1.59	–12.431	<0.001
	Women	1521	3.71	1.55		
2	Men	1456	4.44	1.28	–5.250	<0.001
	Women	1521	4.69	1.13		
3	Men	1456	4.03	1.43	–8.391	<0.001
	Women	1521	4.46	1.30		
4	Men	1456	4.91	1.04	–3.789	<0.001
	Women	1521	5.06	0.95		
5	Men	1456	2.36	1.43	–12.936	<0.001
	Women	1521	3.04	1.52		
6	Men	1456	4.77	1.16	–6.450	<0.001
	Women	1521	5.03	1.03		
7	Men	1456	4.52	1.42	–4.886	<0.001
	Women	1521	4.77	1.31		
8	Men	1456	3.05	1.44	–13.059	<0.001
	Women	1521	3.73	1.39		
9	Men	1456	4.53	1.36	–6.678	<0.001
	Women	1521	4.86	1.19		
10	Men	1456	3.12	1.55	–4.555	<0.001
	Women	1521	3.37	1.48		
11	Men	1456	2.45	1.44	–10.616	<0.001
	Women	1521	3.07	1.61		
12	Men	1456	4.04	1.51	–0.349	0.727
	Women	1521	4.04	1.43		
13	Men	1456	3.75	1.49	–8.967	<0.001
	Women	1521	4.23	1.38		
14	Men	1456	4.30	1.51	–4.128	<0.001
	Women	1521	4.55	1.38		
15	Men	1456	3.89	1.45	–7.076	<0.001
	Women	1521	4.26	1.36		
16	Men	1456	3.75	1.50	–6.538	<0.001
	Women	1521	4.11	1.41		
18	Men	1456	4.80	1.17	–5.491	<0.001
	Women	1521	5.02	1.08		
17	Men	1456	3.76	1.59	–1.480	0.139
	Women	1521	3.85	1.58		
19	Men	1456	3.53	1.62	–6.392	<0.001
	Women	1521	3.91	1.61		
20	Men	1456	4.79	1.43	–7.098	<0.001
	Women	1521	4.47	1.47		

**TABLE 3:** Correlation of the 20 items of the Chinese version of TEPS.

**Item**	**4**	**6**	**18**	**20**	**1**	**5**	**8**	**10**	**11**	**2**	**3**	**7**	**9**	**14**	**12**	**15**	**16**	**17**	**19**	**13**
4	1.000																			
6	0.572^∗∗^	1.000																		
18	0.571^∗∗^	0.581^∗∗^	1.000																	
20	0.331^∗∗^	0.313^∗∗^	0.391^∗∗^	1.000																
1	0.172^∗∗^	0.158^∗∗^	0.179^∗∗^	0.139^∗∗^	1.000															
5	0.097^∗∗^	0.190^∗∗^	0.134^∗∗^	0.116^∗∗^	0.163^∗∗^	1.000														
8	0.147^∗∗^	0.204^∗∗^	0.212^∗∗^	0.140^∗∗^	0.250^∗∗^	0.222^∗∗^	1.000													
10	0.147^∗∗^	0.161^∗∗^	0.227^∗∗^	0.182^∗∗^	0.262^∗∗^	0.204^∗∗^	0.294^∗∗^	1.000												
11	0.088^∗∗^	0.096^∗∗^	0.122^∗∗^	0.117^∗∗^	0.274^∗∗^	0.205^∗∗^	0.271^∗∗^	0.380^∗∗^	1.000											
2	0.476^∗∗^	0.396^∗∗^	0.357^∗∗^	0.238^∗∗^	0.194^∗∗^	0.117^∗∗^	0.144^∗∗^	0.171^∗∗^	0.125^∗∗^	1.000										
3	0.405^∗∗^	0.351^∗∗^	0.315^∗∗^	0.192^∗∗^	0.142^∗∗^	0.188^∗∗^	0.176^∗∗^	0.162^∗∗^	0.119^∗∗^	0.611^∗∗^	1.000									
7	0.403^∗∗^	0.493^∗∗^	0.411^∗∗^	0.246^∗∗^	0.150^∗∗^	0.155^∗∗^	0.258^∗∗^	0.135^∗∗^	0.087^∗∗^	0.393^∗∗^	0.353^∗∗^	1.000								
9	0.383^∗∗^	0.353^∗∗^	0.372^∗∗^	0.239^∗∗^	0.137^∗∗^	0.117^∗∗^	0.198^∗∗^	0.227^∗∗^	0.145^∗∗^	0.417^∗∗^	0.398^∗∗^	0.368^∗∗^	1.000							
14	0.252^∗∗^	0.267^∗∗^	0.321^∗∗^	0.162^∗∗^	0.095^∗∗^	0.101^∗∗^	0.146^∗∗^	0.105^∗∗^	0.080^∗∗^	0.338^∗∗^	0.374^∗∗^	0.335^∗∗^	0.364^∗∗^	1.000						
12	0.229^∗∗^	0.249^∗∗^	0.291^∗∗^	0.215^∗∗^	0.112^∗∗^	0.126^∗∗^	0.185^∗∗^	0.185^∗∗^	0.218^∗∗^	0.261^∗∗^	0.226^∗∗^	0.279^∗∗^	0.248^∗∗^	0.268^∗∗^	1.000					
15	0.262^∗∗^	0.291^∗∗^	0.335^∗∗^	0.217^∗∗^	0.182^∗∗^	0.164^∗∗^	0.310^∗∗^	0.226^∗∗^	0.159^∗∗^	0.270^∗∗^	0.273^∗∗^	0.292^∗∗^	0.286^∗∗^	0.344^∗∗^	0.308^∗∗^	1.000				
16	0.228^∗∗^	0.253^∗∗^	0.321^∗∗^	0.214^∗∗^	0.181^∗∗^	0.159^∗∗^	0.311^∗∗^	0.233^∗∗^	0.170^∗∗^	0.236^∗∗^	0.234^∗∗^	0.249^∗∗^	0.237^∗∗^	0.262^∗∗^	0.279^∗∗^	0.612^∗∗^	1.000			
17	0.206^∗∗^	0.249^∗∗^	0.352^∗∗^	0.195^∗∗^	0.069^∗∗^	0.167^∗∗^	0.162^∗∗^	0.125^∗∗^	0.096^∗∗^	0.239^∗∗^	0.305^∗∗^	0.294^∗∗^	0.282^∗∗^	0.387^∗∗^	0.311^∗∗^	0.325^∗∗^	0.368^∗∗^	1.000		
19	0.209^∗∗^	0.231^∗∗^	0.286^∗∗^	0.287^∗∗^	0.175^∗∗^	0.259^∗∗^	0.217^∗∗^	0.217^∗∗^	0.210^∗∗^	0.240^∗∗^	0.216^∗∗^	0.213^∗∗^	0.262^∗∗^	0.203^∗∗^	0.224^∗∗^	0.253^∗∗^	0.268^∗∗^	0.279^∗∗^	1.000	
13	0.141^∗∗^	0.150^∗∗^	0.132^∗∗^	0.065^∗∗^	0.114^∗∗^	–0.003	0.101^∗∗^	0.038^∗^	–0.011	0.076^∗∗^	0.020	0.104^∗∗^	0.064^∗∗^	–0.028	–0.013	0.083^∗∗^	0.093^∗∗^	–0.031	0.045^∗^	1.000

*Item 13 had no or very low correlations with other items as was the case in Chan et al.’s (2012a) study, and was discarded in further analyses. ^∗^*p* < 0.05. ^∗∗^*p* < 0.01.*

**TABLE 4:** Correlations between factors in the total sample and sub group.

	**AA**	**CA**	**AC**	**CC**
**The total**				
AA	1			
CA	0.481^∗∗^	1		
AC	0.711^∗∗^	0.518^∗∗^	1	
CC	0.560^∗∗^	0.638^∗∗^	0.617^∗∗^	1
**Men**				
AA	1			
CA	0.389^∗∗^	1		
AC	0.692^∗∗^	0.453^∗∗^	1	
CC	0.531^∗∗^	0.626^∗∗^	0.621^∗∗^	1
**Women**				
AA	1			
CA	0.556^∗∗^	1		
AC	0.732^∗∗^	0.528^∗∗^	1	
CC	0.589^∗∗^	0.636^∗∗^	0.605^∗∗^	1

*AA, abstract anticipatory pleasure; CA, contextual anticipatory pleasure; AC, abstract consummatory pleasure; CC, contextual consummatory pleasure; ANT, anticipatory pleasure; CON, consummatory pleasure. All correlations with “^∗∗^” are statistically significant at *p* ≤ 0.01 level.*

### Single Group Confirmatory Factor Analyses

To determine which was the better fitting model, the hypothesized four-factor model was compared with the two-factor model by using CFI and Bayes information criterion indices (the model with the bigger CFI and smaller Bayes information criterion values is the better fitting one). The fit indices of the two-factor model were as follows: χ^2^(df = 151; *p* < 0.001) = 3093.005; CFI = 0.756; TLI = 0.723; RMSEA = 0.081 with an interval at 90% (0.078–0.083); and BIC = 18,7041.51. While Goodness of fit indices of the four-factor model were found as χ^2^(df = 146; *p* < 0.001) = 1563.794; CFI = 0.882; TLI = 0.862; RMSEA = 0.057 with an interval at 90% (0.055–0.060); and BIC = 18,5231.48. Because of the bigger CFI and smaller Bayes information criterion values of the four-factor model, the four-factor model was better than the two-factor model. As presented in Table 5, the standardized factor loadings of each item for the four-factor model of the TEPS were all above 0.30, ranging from 0.385 to 0.764.

**TABLE 5:** Factor loadings of the 4-factor solution for the TEPS in the Chinese sample.

**Items**		**Standardized factor loading**			
		
		**AA**	**CA**	**AC**	**CC**
4	I look forward to a lot of things in my life	0.749			
6	Looking forward to a pleasurable experience is in itself pleasurable	0.734			
18	When something exciting is coming up in my life, I really look forward to it	0.764			
20	On the way to my first date with my beloved, I can hardly wait to see him/her	0.442			
1	When I hear about a new movie starring my favorite actor, I can’t wait to see it		0.462		
5	I love it when people play with my hair		0.385		
8	When I think of something tasty, like a chocolate chip cookie, I have to have one		0.566		
10	I get so excited the night before a major holiday I can hardly sleep		0.582		
11	When I’m on my way to an amusement park, I can hardly wait to ride the roller coasters		0.535		
2	I enjoy taking a deep breath of fresh air when I walk outside			0.720	
3	The smell of freshly cut grass is enjoyable to me			0.695	
7	A hot cup of coffee or tea on a cold morning is very satisfying to me			0.544	
9	I appreciate the beauty of a fresh snowfall			0.598	
14	I love the sound of rain on the windows when I’m lying in my warm bed			0.494	
12	I really enjoy the feeling of a good yawn				0.465
15	When I think about eating my favorite food, I can almost taste how good it is				0.728
16	When ordering something off the menu, I imagine how good it will taste				0.722
17	The sound of crackling wood in the fireplace is very relaxing				0.515
19	I love it when a baby snuggles into my arms				0.440
13	I don’t look forward to things like eating out at restaurants				

*AA, abstract anticipatory pleasure; CA, contextual anticipatory pleasure; AC, abstract consummatory pleasure; CC, contextual consummatory pleasure.*

As presented in Table 6, in the total sample, results of the CFA showed that the four-factor model was narrowly acceptable although the CFI was slightly lower than 0.9. The modifications included adding correlations of the error variance between item 16 and item 17 of Contextual Consummatory (“When ordering something off the menu, I imagine how good it will taste” and “The sound of crackling wood in the fireplace is very relaxing”) and item 10 and item 11 of Contextual Anticipatory (“I get so excited the night before a major holiday I can hardly sleep” and “When I’m on my way to an amusement park, I can hardly wait to ride the roller coasters”)^1^. These modifications were decided based on the modification indices in Mplus output and consideration of substantive meaningfulness. Measurement error covariances between two items may be due to similar wording, content, or directionality. This situation applies to our case, occurring when multiple items have a degree of overlap in content and assess the same construct (Byrne, 2012). Thus, this study correlated these error terms and the fit of the model was improved considerably to acceptable. Similarly, initial CFA models for the TEPS were not acceptable in men and women. After the same items were modified, the fitting of the model also reached the proposed standard. In sum, the findings supported that the four-factor model was superior to the two-factor model. And the present study retained the four-factor model (with two error correlations) as the final one.

**TABLE 6:** Goodness-of-fit indices for each model in total sample and sub group.

**Model**	**χ^2^**	**df**	***p***	**CFI**	**TLI**	**RMSEA (90%CI)**	**BIC**
**The total sample**							
Two-factor model	3093.005	151	<0.001	0.756	0.723	0.081(0.078–0.083)	18, 7041.51
Four-factor model	1563.794	146	<0.001	0.882	0.862	0.057(0.055–0.060)	18, 5231.48
Four-factor model with two error correlations	1054.388	144	<0.001	0.924	0.910	0.046(0.043–0.049)	18, 4622.87
**Males**							
Four-factor model	745.801	146	<0.001	0.896	0.878	0.053(0.049–0.057)	91, 653.65
Four-factor model with two error correlations	542.570	144	<0.001	0.931	0.918	0.044(0.040–0.048)	91, 423.45
**Females**							
Four-factor model	1084.989	146	<0.001	0.844	0.817	0.065(0.061–0.068)	93, 805.27
Four-factor model with two error correlations	736.184	144	<0.001	0.901	0.883	0.052(0.048–0.056)	93, 385.42

*χ^2^, chi-square; df, degrees of freedom; CFI, comparative fit index; TLI, Tucker–Lewis index; BIC, Bayesian information criterion; RMSEA, root mean square error of approximation; CI, confidence interval.*

### Measurement Invariance Across Gender

Measurement invariance evaluation was started with the model that had no gender-invariance constraints. As can be seen in Table 7, although TLI of the configural model was a little lower than 0.9, the rest of indices all met the requirements (χ^2^(288) = 1282.908, *p* < 0.001, CFI = 0.915, RMSEA = 0.048), suggesting that the configural invariance was supported. This configural model was then used as the baseline model to compare against the more restrictive measurement invariance models.

**TABLE 7:** Fit statistics for measurement invariance models across gender.

**Model**	**χ^2^**	**df**	***p***	**CFI**	**TLI**	**RMSEA (90%CI)**	**ΔCFI**	**ΔTFI**	**BIC**
Configural	1282.908	288	<0.001	0.915	0.899	0.048(0.046–0.051)			1,83,648.361
Metric	1317.142	303	<0.001	0.913	0.902	0.047(0.045–0.050)	−0.002	0.003	1,83,561.571
Scalar	1571.874	318	<0.001	0.893	0.885	0.052(0.049–0.054)	−0.020	–0.017	1,83,738.359
Partial scalar	1405.580	316	<0.001	0.907	0.899	0.048(0.046–0.051)	−0.006	0.003	1,83,554.388

*χ^2^, chi-square; df, degrees of freedom; CFI, comparative fit index; TLI, Tucker–Lewis index; BIC, Bayesian information criterion; RMSEA, root mean square error of approximation; CI, confidence interval; Δ, change in the parameter.*

Then the metric invariance model was examined, with item loadings being treated as invariant for men and women. When compared against the less-constrained models, the results of ΔCFI = 0.002 and a decrease of 86.79 in BIC value implied support for metric invariance across gender. Accordingly, the same item of the Chinese TEPS represents the same concept across university students with different gender.

On the basis of the previous two steps, the item intercepts were further constrained to be equal between men and women. However, this restriction resulted in worse model fit, because a ΔCFI = 0.02 was bigger than 0.01, and also improvements in BIC value was found. In sum, results above did not support full scalar invariance across gender. Therefore, we conducted further analyses to detect whether equal intercept constrain on some items could be released to obtain a partial scalar invariance model. Inspecting intercept differences on observed items showed that item 4 of abstract anticipatory pleasure and item 8 of contextual anticipatory pleasure had contributed to the significant change in the CFI value. As a result, this study examined a partial scalar model in which intercepts of all the items were fixed equal except item 4 and 8 were free to vary across groups. According to no significant worse on CFI and decrease on BIC from the metric model, partial scalar invariance established.

Finally, comparisons of factor means could be tested with no need for controlling non-invariance. In this study, the male group served as the reference group and factor means among male were constrained to zero. We tested the factor means difference by examining the estimated values of the latent means among the female with its standard error. On the basis of the partial scalar model, standardized factor mean differences between men and women were 6.072 (*p* < 0.001) on Abstract Anticipatory, 16.724 (*p* < 0.001) on Contextual Anticipatory, 8.405 (*p* < 0.001) on Abstract Consummatory, and 6.242 (*p* < 0.001) on Abstract Consummatory. Gender difference on the four factors were statistically significant, and men had lower levels on all of the four factors compared with women.

## Discussion

The current study confirmed the factor structure, examined the measurement invariance of the TEPS in a sample of Chinese young adults, and explored gender difference on latent factors. This investigation contributes to the empirical research on the TEPS by providing the first evidence for the measurement invariance across gender.

First of all, results of our study are in accord with previous findings, showing that the TEPS has a four-factor structure in Chinese sample, which is better than alternative two-factor solutions (Chan et al., 2012a; Li Z. et al., 2015; Li et al., 2018). And the revised four-factor model of the TEPS provided an adequate fit to the data in the full sample as well as in each gender group. Chan et al.’s (2012a) study pointed out that the labels of “abstract” versus “contextual” appear appropriate for the anticipatory items, but do not seem to fit as well in distinguishing the two consummatory factors. In other words, the items on the “abstract” consummatory factor may refer to specific contextual pleasures and the idea of a consummatory pleasure may be also inherently contextual. Thus, abstract consummatory factor was positively and highly correlated with contextual consummatory factor, while correlation coefficient between abstract anticipatory factor and contextual anticipatory factor was relatively low. Consistent with the study of Chan et al. (2012a), the correlation between abstract anticipatory factor and abstract consummatory factor was stronger than that between abstract anticipatory factor and contextual anticipatory, which emphasized the importance of parsing anticipatory pleasure into abstract and contextual component. So did consummatory pleasure. It’s also necessary to classify consummatory pleasure into abstract and contextual component, given that the correlation between contextual anticipatory factor and contextual consummatory was stronger than between abstract consummatory factor and contextual consummatory factor.

Further analysis on measurement invariance tests suggested full configural and full metric invariance, and partial scalar invariance of the TEPS across gender. That is to say, the four-factor structure of the scale holds both in men and woman, the factor loading of each observed item on corresponding latent variable is equal between different sex groups, and the same degree of change of observed variable have the same meaning regardless of gender.

The determination of the scalar invariance suggested that the intercepts of each variable are invariant and have the same reference points across groups. However, the adapted TEPS generally did not convincingly support the full scalar invariance for gender. There existed two items (item 4 and 8) whose intercepts weren’t invariant across different gender groups. Women scored higher than men on both items. Item 4 (I look forward to a lot of things in my life) is about the pleasure anticipation, which is related to the future goals. Greene and DeBacker (2004) found that females have more future goals than males, which may help explain our results. Item 8 (When I think of something tasty, like a chocolate chip cookie, I have to have one) is related to the pleasure feelings responding to the sweets. Women with higher scores on item 8 in present study might be consistent with the previous findings that women appreciated sweetness more than males did (Katou et al., 2005; Sena-Esteves et al., 2018). Notably, no studies have examined the measurement invariance of the TEPS across gender in other cultural contexts, so it is not clear whether the non-invariant intercepts of item 4 and 8 exist only in Chinese male and female, which warrants future exploration. Our results supported full metric and partial scalar invariance which suggested that gender differences of pleasure experience could be reflected by comparing latent means but not the observed scores (Cheung and Rensvold, 2002). It worth noting that if gender differences were reported on item 4 and 8, researchers should be careful whether it could be attributed to authentic differences between genders or to measurement differences across gender because of the non-invariant intercepts of item 4 and 8.

On the four dimensions of the TEPS, our findings revealed that men reported lower levels of pleasure compared to women. In other words, men presented higher level of anhedonia, which was consistent with previous studies (Miettunen et al., 2010; Gooding and Pflum, 2014; Gooding et al., 2017; Jha et al., 2018). On abstract anticipatory pleasure, there was no significant difference of the observed means between men and women, whereas women had higher latent means than men. By reason of partial scalar invariance supported in this study, the mean difference of latent variables between groups cannot be represented by observed mean scores (Tucker et al., 2006). Thus, the difference in observed means might not represent the real difference across gender, which means it’s inappropriate to compare gender difference based on item scores without confirmation of measurement invariance. And this is exactly the significance of testing measurement invariance.

One of limitations in the present study was that we used a convenience sample of students from universities, so it remains to be determined whether the results could be generalizable to other adult groups who have different education level. Having no other related measures to analyze the construct validity of the instrument is another limitation of this study. This study examined the measurement invariance across gender of the Chinese version of the TEPS, but it still unknown whether other language versions of the TEPS are measurement invariant across gender. Future researches exploring factorial invariance of different language versions of the TEPS across gender are welcomed. In addition, the TEPS can be used in both clinical patients and healthy people. In this study, only undergraduates were included and no clinical samples were involved. Thus, measurement invariance of the TEPS across clinical status should be examined in the future.

## Conclusion

The results of this study indicate that the presently developed four-factor model better represented the underlying structure of the TEPS in Chinese samples. Evidence supported configural, metric and partial scalar invariance of the TEPS across gender in a large sample of Chinese university students. And males had significant higher level of anhedonia than females. The evidence of partial rather than full scalar invariance suggests that difference comparisons should be conducted not using observed scores but latent means cautiously.

## Data Availability

The raw data supporting the conclusions of this manuscript will be made available by the authors, without undue reservation, to any qualified researcher.

## Ethics Statement

The studies involving human participants were reviewed and approved by the Ethics Committee of the Second Xiangya Hospital of Central South University. The patients/participants provided their written informed consent to participate in this study.

## Author Contributions

HZ conceived and designed the study. XZ had full access to all the data in the study and took responsibility for the integrity of the data and the accuracy of the data analysis. JX and JZ collected and inputted data. HZ, WL, and XZ contributed to interpret the data. HZ wrote the first draft of the manuscript. HZ and JF revised the manuscript. All authors read and approved the final version.

## Conflict of Interest Statement

The authors declare that the research was conducted in the absence of any commercial or financial relationships that could be construed as a potential conflict of interest.

## Footnotes

1 Error correlation coefficient was −0.137 (*p* < 0.05) between item 16 and item 17, and −0.143 (*p* < 0.05) between item 10 and item 11. In this study, items 16 and item 17 both focus on in-the-moment experience in response to a specific sensory stimulus, and reflect Contextual Consummatory, while items 11 and item 10 both measure the pleasure experienced in anticipation of a positive or pleasurable stimulus and the response facing positive or pleasurable stimulus, and reflect Contextual Anticipatory. In the other word, item 16 and item 17 were worded similarly and assess similar content, which might create a residual correlation between them. This explanation also applies to the residual correlation between item 10 and item 11.

## References

[B1] Baumgartner H. Steenkamp J.-B. E. M. (1998). Assessing measurement invariance in cross-national consumer research. *J. Consum. Res.* 25 78–90. 10.1086/209528

[B2] Berridge K. C. Kringelbach M. L. (2008). Affective neuroscience of pleasure: reward in humans and animals. *Psychopharmacology* 199 457–480. 10.1007/s00213-008-1099-6 18311558 PMC3004012

[B3] Berridge K. C. Robinson T. E. (1998). What is the role of dopamine in reward: hedonic impact, reward learning, or incentive salience? *Brain Res. Brain Res. Rev.* 28 309–369. 10.1016/s0165-0173(98)00019-8 9858756

[B4] Berridge K. C. Robinson T. E. (2003). Parsing reward. *Trends Neurosci.* 26 507–513. 10.1016/s0166-2236(03)00233-9 12948663

[B5] Brown T. A. (2006). *Confirmatory Factor Analysis for Applied Research.* New York, NY: Guilford Press.

[B6] Byrne B. M. (2012). *Structural Equation Modeling with Mplus: Basic Concepts, Applications, and Programming.* New York, NY: Routledge, Taylor & Francis.

[B7] Carver C. S. White T. L. (1994). Behavioral inhibition, behavioral activation, and affective responses to impending reward and punishment: the BIS/BAS scales. *J. Pers. Soc. Psychol.* 67 319–333. 10.1037//0022-3514.67.2.319

[B8] Chan R. C. K. Shi Y.-F. Lai M.-K. Wang Y.-N. Wang Y. Kring A. M. (2012a). The temporal experience of pleasure scale (TEPS): exploration and confirmation of factor structure in a Healthy Chinese sample. *PLoS One* 7:e35352. 10.1371/journal.pone.0035352 22530007 PMC3329425

[B9] Chan R. C. K. Wang Y. Yan C. Zhao Q. McGrath J. Hsi X. (2012b). A study of trait anhedonia in non-clinical chinese samples: evidence from the chapman scales for physical and social anhedonia. *PLoS One* 7:e34275. 10.1371/journal.pone.0034275 22529910 PMC3328477

[B10] Chen F. F. (2007). Sensitivity of goodness of fit indexes to lack of measurement invariance. *Struct. Equ. Model. Multidiscipl. J.* 14 464–504. 10.1080/10705510701301834

[B11] Cheung G. W. Rensvold R. B. (2002). Evaluating goodness-of-fit indexes for testing measurement invariance. *Struct. Equ. Model. Multidiscipl. J.* 9 233–255. 10.1207/s15328007sem0902_5 22551991

[B12] Da Silva S. Saperia S. Siddiqui I. Fervaha G. Agid O. Daskalakis Z. J. (2017). Investigating consummatory and anticipatory pleasure across motivation deficits in schizophrenia and healthy controls. *Psychiatry Res.* 254 112–117. 10.1016/j.psychres.2017.04.040 28460280

[B13] Dietrich A. Ormel J. Buitelaar J. K. Verhulst F. C. Hoekstra P. J. Hartman C. A. (2013). Cortisol in the morning and dimensions of anxiety, depression, and aggression in children from a general population and clinic-referred cohort: an integrated analysis. The TRAILS study. *Psychoneuroendocrinology* 38 1281–1298. 10.1016/j.psyneuen.2012.11.013 23237815

[B14] Gard D. E. Gard M. G. Kring A. M. John O. P. (2006). Anticipatory and consummatory components of the experience of pleasure: a scale development study. *J. Res. n Pers.* 40 1086–1102. 10.1016/j.jrp.2005.11.001 29431282

[B15] Geaney J. T. Treadway M. T. Smillie L. D. (2015). Trait anticipatory pleasure predicts effort expenditure for reward. *PLoS One* 10:e0131357. 10.1371/journal.pone.0131357 26115223 PMC4482634

[B16] Gooding D. C. Chan R. C. K. Zhou H.-Y. Li Z. Cheung E. F. C. (2017). The indirect assessment of social anhedonia in Chinese adolescents: preliminary findings. *Psychiatry Res.* 257 418–423. 10.1016/j.psychres.2017.08.007 28837930

[B17] Gooding D. C. Pflum M. J. (2014). Further validation of the ACIPS as a measure of social hedonic response. *Psychiatry Res.* 215 771–777. 10.1016/j.psychres.2013.11.009 24393478

[B18] Gooding D. C. Pflum M. J. Fonseca-Pedero E. Paino M. (2016). Assessing social anhedonia in adolescence: the ACIPS-A in a community sample. *Eur. Psychiatry* 37 49–55. 10.1016/j.eurpsy.2016.05.012 27447103

[B19] Greene B. A. DeBacker T. K. (2004). Gender and orientations toward the future: links to motivation. *Educ. Psychol. Rev.* 16 91–120. 10.1023/B:EDPR.0000026608.50611.b4

[B20] Gross J. J. John O. P. (1995). Facets of emotional expressivity: three self-report factors and their correlates. *Pers. Indiv. Diff.* 19 555–568. 10.1016/0191-8869(95)00055-b

[B21] Harvey P. O. Pruessner J. Czechowska Y. Lepage M. (2007). Individual differences in trait anhedonia: a structural and functional magnetic resonance imaging study in non-clinical subjects. *Mol. Psychiatry* 12 767–775. 10.1038/sj.mp.4002021 17505465

[B22] Ho P. M. Cooper A. J. Hall P. J. Smillie L. D. (2015). Factor structure and construct validity of the temporal experience of pleasure scales. *J. Pers. Assess.* 97 200–208. 10.1080/00223891.2014.940625 25101907

[B23] Hollenbeck J. R. Klein H. (1987). Goal commitment and the goal-setting process: problems, prospects, and proposals for future research. *J. Appl. Psychol.* 72 212–220. 10.1037//0021-9010.72.2.212

[B24] Hu L.-T. Bentler P. M. (1995). “Evaluating model fit,” in *Structural Equation Modeling: Concepts, Issues, and Applications*, ed. Hoyle R. H. (Thousand Oaks, CA: Sage Publications, Inc), 76–99.

[B25] Jha M. K. Miller A. H. Minhajuddin A. Trivedi M. H. (2018). Association of T and non-T cell cytokines with anhedonia: role of gender differences. *Psychoneuroendocrinology* 95 1–7. 10.1016/j.psyneuen.2018.05.017 29783087 PMC6312182

[B26] Katou Y. Mori T. Ikawa Y. (2005). Effect of age and gender on attitudes towards sweet foods among Japanese. *Food Qual. Prefer.* 16 171–179. 10.1016/j.foodqual.2004.04.012

[B27] Kelley M. E. van Kammen D. P. Allen D. N. (1999). Empirical validation of primary negative symptoms: independence from effects of medication and psychosis. *Am. J. Psychiatry* 156 406–411. 10080556 10.1176/ajp.156.3.406

[B28] Keshavan M. S. DeLisi L. E. Seidman L. J. (2011). Early and broadly defined psychosis risk mental states. *Schizophrenia Res.* 126 1–10. 10.1016/j.schres.2010.10.006 21123033 PMC3388534

[B29] Kline B. R. (2010). *Principles and Practice of Structural Equation Modeling.* New York, NY: Guilford Press.

[B30] Kringelbach M. L. Berridge K. C. (2009). Towards a functional neuroanatomy of pleasure and happiness. *Trends Cogn. Sci.* 13 479–487. 10.1016/j.tics.2009.08.006 19782634 PMC2767390

[B31] Li S. Zhang Y. Fan J. Liu W. Gan J. He J. (2019). Patients with obsessive-compulsive disorder exhibit deficits in consummatory but not anticipatory pleasure. *Front. Psychol.* 10:1196. 10.3389/fpsyg.2019.01196 31231272 PMC6558405

[B32] Li Y. H. Mou X. D. Jiang W. H. Yang Z. Shen X. H. Jin Z. M. (2015). A comparative study of anhedonia components between major depression and schizophrenia in Chinese populations. *Ann. Gen. Psychiatry* 14:24. 10.1186/s12991-015-0061-3 26339277 PMC4558731

[B33] Li Z. Lui S. S. Y. Geng F.-L. Li Y. Li W.-X. Wang C.-Y. (2015). Experiential pleasure deficits in different stages of schizophrenia. *Schizophrenia Res.* 166 98–103. 10.1016/j.schres.2015.05.041 26072322

[B34] Li Z. Shi H.-S. Elis O. Yang Z.-Y. Wang Y. Lui S. S. Y. (2018). The structural invariance of the temporal experience of pleasure scale across time and culture. *Psych J.* 7 59–67. 10.1002/pchj.207 29431282

[B35] Little T. D. Card N. A. Slegers D. W. Ledford E. C. (2007). “Representing contextual effects in multiple-group MACS models,” in *Modeling Contextual Effects in Longitudinal Studies*, eds Little T. D. Bovaird J. A. Card N. A. (Mahwah, NJ: Lawrence Erlbaum Associates Publishers), 121–147.

[B36] Miettunen J. Veijola J. Freimer N. Lichtermann D. Peltonen L. Paunio T. (2010). Data on schizotypy and affective scales are gender and education dependent — study in the Northern Finland 1966 Birth Cohort. *Psychiatry Res.* 178 408–413. 10.1016/j.psychres.2008.07.022 20478630

[B37] Miller L. S. Burns S. A. (1995). Gender differences in Schizotypic features in a large sample of young adults. *J. Nervous Ment. Dis.* 183 657–661. 10.1097/00005053-199510000-00007 7561812

[B38] Mote J. Minzenberg M. J. Carter C. S. Kring A. M. (2014). Deficits in anticipatory but not consummatory pleasure in people with recent-onset schizophrenia spectrum disorders. *Schizophrenia Res.* 159 76–79. 10.1016/j.schres.2014.07.048 25139112 PMC4177285

[B39] Muthén L. K. Muthén B. O. (1998–2012.) *Mplus, Version 7.3. Computer software.* Los Angeles, CA: Muthén and Muthén. 10.1016/j.schres.2014.07.048

[B40] Novacek D. M. Gooding D. C. Pflum M. J. (2016). Hedonic capacity in the broader autism phenotype: should social anhedonia be considered a characteristic feature? *Front. Psychol.* 7:666. 10.3389/fpsyg.2016.00666 27199879 PMC4858588

[B41] Oishi S. Schimmack U. Diener E. (2001). Pleasures and subjective well-being. *Eur. J. Pers.* 15 153–167. 10.1002/per.406

[B42] Petrowski K. Kliem S. Sadler M. Meuret A. E. Ritz T. Brähler E. (2018). Factor structure and psychometric properties of the english version of the trier inventory for chronic stress (TICS-E). *BMC Med. Res. Methodol.* 18:18. 10.1186/s12874-018-0471-4 29409461 PMC5801899

[B43] Rossi G. Elklit A. Simonsen E. (2010). Empirical evidence for a four factor framework of personality disorder organization: multigroup confirmatory factor analysis of the Millon clinical multiaxial inventory - III personality disorder scales across Belgian and Danish data samples. *J. Pers. Disord.* 24 128–150. 10.1521/pedi.2010.24.1.128 20205502

[B44] Seidlitz L. Diener E. (1998). Sex differences in the recall of affective experiences. *J. Pers. Soc. Psychol.* 74 262–271. 10.1037//0022-3514.74.1.262 9457787

[B45] Sena-Esteves M. M. Mota M. Malfeito-Ferreira M. (2018). Patterns of sweetness preference in red wine according to consumer characterisation. *Food Res. Int.* 106 38–44. 10.1016/j.foodres.2017.12.043 29579938

[B46] Simon J. J. Zimmermann J. Cordeiro S. A. Maree I. Gard D. E. Friederich H.-C. (2018). Psychometric evaluation of the temporal experience of pleasure scale (TEPS) in a German sample. *Psychiatry Res.* 260 138–143. 10.1016/j.psychres.2017.11.060 29195165

[B47] Tucker K. L. Ozer D. J. Lyubomirsky S. Boehm J. K. (2006). Testing for measurement invariance in the satisfaction with life scale: a comparison of Russians and North Americans. *Soc. Indic. Res.* 78 341–360. 10.1007/s11205-005-1037-5

[B48] van de Schoot R. Lugtig P. Hox J. (2012). A checklist for testing measurement invariance. *Eur. J. Dev. Psychol.* 9 486–492. 10.1080/17405629.2012.686740

[B49] Vandenberg R. J. Lance C. E. (2000). A review and synthesis of the measurement invariance literature: suggestions, practices, and recommendations for organizational research. *Organ. Res. Methods* 3 4–70. 10.1177/109442810031002

[B50] Wu H. Mata J. Furman D. J. Whitmer A. J. Gotlib I. H. Thompson R. J. (2017). Anticipatory and consummatory pleasure and displeasure in major depressive disorder: an experience sampling study. *J. Abnorm. Psychol.* 126 149–159. 10.1037/abn0000244 27936838 PMC5305427

[B51] Yang Z. Y. Xie D. J. Zou Y. M. Wang Y. Li Y. Shi H. S. (2018). Prospection deficits in schizophrenia: evidence from clinical and subclinical samples. *J. Abnorm. Psychol.* 127 710–721. 10.1037/abn0000382 30335440

